# Relation between mortality for four years and computerized tomography by marshall score in traumatic brain injury critical patients

**DOI:** 10.1186/2197-425X-3-S1-A373

**Published:** 2015-10-01

**Authors:** M Delange-Van Der Kroff, E Aguilar-Alonso, MD Arias-Verdu, E Curiel-Balsero, A Muñoz-Lopez, F Fernandez-Ortego, C De la Fuente-Martos, R Rivera-Fernandez, MA Prieto-Palomino

**Affiliations:** Intensive Care, Axarquía Hospital, Velez-Malaga, Spain; Intensive Care, Infanta Margarita Hospital, Cabra, Spain; Intensive Care, Regional University Hospital, Malaga, Spain

## Introduction

Many instruments have been developed to evaluate hospital mortality, but less attention has been paid to long-term mortality of traumatic brain injury patients.

## Objectives

To analyze the relationship between type of injury used computerized tomography (CT) by Marshall score on admission and mortality at four years in traumatic brain injury critical patients admitted to intensive care.

## Methods

Prospective cohort study of traumatic brain injury patients admitted in the University Clinical Hospital (Malaga) between 2004 to 2008. Data were collected to calculate different prognostic scores and cranial CT at admission by Marshall score.

The results were analyzed at discharge, at one and four years. T-test, X^2^ and multiple logistic regression. P < 0.05 was considered significant.

## Results

531 adult patients. Mean age 40.35 ± 19.75 years. At first year, 171 patients had died (32.2%): 133 in intensive care (25%), 19 in hospital (hospital mortality 28.6%) and 19 out of the hospital to follow up; 35 missing cases (6.6%). At 4 years, 181 had died (34.1%); 86 cases were lost to follow up (16.2%).

The patients dead at four years to follow up were older (48.68 ± 21.58 vs 34.45 ± 16.99 years; p < 0.001), more severity by APACHE-II (23.12 ± 6.38 vs 14.88 ± 5.57 points, p < 0.001) and higher admission coma degree (5.59 ± 3.28 vs 8.62 ± 3.61 points, p < 0.001).

Mortality at 4 years is associated with type of injury used computerized tomography (CT) by Marshall score on admission. Mortality at diffuse injury type I was 9.1% vs 68.2% in injury type IV.

By logistic regression the mortality at 4 years was associated with APACHE-II (OR: 1.12; CI 1.06-1.18), age (OR: 1.04, CI 1.03-1.06), Injury Severity Score (OR: 1.02; CI 1-1.04), Glasgow coma scale at admission (OR: 0.84, CI 0.76-0.93), tracheostomy (OR: 0.21; IC 0,12-0,37) and Marshall classification: diffuse injury type I (OR: 1), type II (OR: 1.48; CI 0,46-4.77), type III (OR: 2.94; CI 0.94-9.16) and type IV (OR: 9.97, CI 2.68-37.54); evacuated mass (OR: 4.6; CI 1.45-42.65), not evacuated mass (OR: 10.07; IC 2,37-42,65).

## Conclusions

The type of injury at admission CT in traumatic brain injury patients admitted to ICU and evaluated by Marshall score is related to mortality at 4 years. Mortality after the first year is very low.

